# Migrated biliary stent into the portal vein: a case report

**DOI:** 10.1093/jscr/rjae065

**Published:** 2024-02-13

**Authors:** Sanne H Christensen, Nawal F Roga, Jakob Kirkegaard, Jacob Nygaard

**Affiliations:** Department of Surgery, University Hospital Aarhus, Palle Juul-Jensens Boulevard 99, 8200 Aarhus N, Denmark; Department of Surgery, University Hospital Aarhus, Palle Juul-Jensens Boulevard 99, 8200 Aarhus N, Denmark; Department of Surgery, University Hospital Aarhus, Palle Juul-Jensens Boulevard 99, 8200 Aarhus N, Denmark; Department of Surgery, University Hospital Aarhus, Palle Juul-Jensens Boulevard 99, 8200 Aarhus N, Denmark

**Keywords:** ERCP, biliary stent, migration, biliary stent complications

## Abstract

Biliary stent insertion is a well-established treatment of bile duct obstruction. Stent migration is a late-onset complication and can be life-threatening. We present a case of proximal biliary stent migration to the portal vein. An upper endoscopy was performed, and the biliary stent was retracted. Bleeding was seen from the papilla of Vater. The hepatoduodenal ligament was explored, and the common bile duct was found fully adherent to the portal vein, with a fistula between the common bile duct and the portal vein. The patient underwent surgery with extrahepatic bile duct resection and hepaticoduodenostomy. The patient survived and was discharged without surgical postoperative complications on postoperative Day 8.

## Introduction

Endoscopic biliary stent insertion is a well-established treatment of benign or malignant biliary duct obstruction [1]. Migration of biliary stents is a well-known and well-described late-onset complication [2–4]. Prior research found a risk of up to 6% for biliary stent migration. While distal migration is the most common, proximal migration is a more serious complication [5, 6]. We present a rare case of proximal migration of a biliary stent into the portal vein.

## Case report

A 72-year-old female presented with 9 days of right-sided abdominal pain and fever (39.3°C) to a local emergency department. On abdominal examination, she had diffuse pain upon palpation, with the point of maximum in upper left and right quadrant, non-peritoneal. Blood tests showed elevated levels of C-reactive protein (CRP) 113 mg/L, total bilirubin 110 mg/L, and alkaline phosphatase (ALP) 450 IU/L. Leukocytes level was normal. An ultrasound of the abdomen was performed with no positive findings. A computed tomography (CT) scan revealed a gallbladder with sludge, stone, and wall thickening. The following day an endoscopic retrograde cholangiopancreatography (ERCP) was conducted due to increasing bilirubin levels 110 to 257 mg/L and biochemical sign of infection (CRP increasing to 144 mg/L). The ERCP showed a normal cholangiogram without any obstructions. Despite absence of biliary duct obstruction, a plastic stent (8,5 French 7 cm), was placed in the common bile duct. The patient was discharged on postoperative Day 2 with scheduled outpatient visit after 1 week. 6 days after discharge the patient experienced worsening abdominal pain. A magnetic resonance cholangiopancreatography revealed stones in the gallbladder but no stones in the common bile duct. Again, the abdominal pain subsided, and the initial plan with stent removal after 8–12 weeks was maintained.

**Figure 1:**
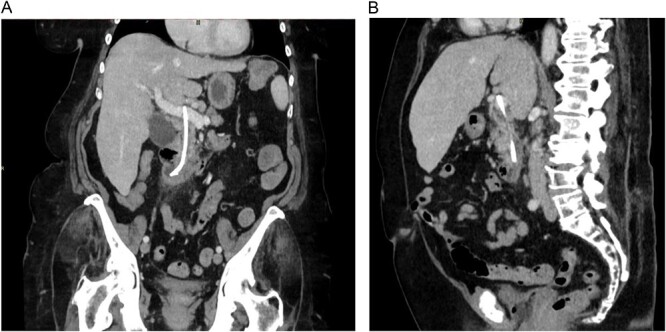
CT images showing the migrated common bile duct stent in the portal vein. (A) Coronal view. (B) Sagittal view.

One month later, the patient once again presented to the local emergency department with fever (39.5°C) and pain in the upper right quadrant. Blood tests showed sign of cholangitis with CRP of 144 mg/L and total bilirubin of 43 mg/L. An abdominal CT was performed and stent migration into the portal vein was suspected (Fig. 1).

Due to the risk of bleeding from the portal vein, the patient was transferred to a hepato-pancreato-biliary center. Overnight, hemoglobin levels dropped from 6.2 to 5.7 mmol/L. The patient refused any kind of transfusions. After multidisciplinary team assessment, endoscopic stent removal with surgical backup was chosen.

An upper endoscopy was performed, and the biliary stent was retracted. After a period of observation, bleeding was seen from the papilla of Vater. A laparotomy and cholecystectomy was performed. The hepatoduodenal ligament was explored, and it was noted that the common bile duct was fully adherent to the portal vein, and a fistula between the common bile duct and the portal vein was found. The extrahepatic bile ducts were resected, the fistula was closed, and a hepaticoduodenostomy was constructed. The patient had an uneventful recovery and was discharged on postoperative Day 8.

## Discussion

Proximal biliary stent migration is a rare complication and can be life-threatening [4]. To our knowledge, only a few cases with migration to the portal vein have been reported [3, 4]. Bleeding from a fistula to the portal vein tends to be self-limiting due to the low blood pressure in the portal system [3]. In this case, bleeding was expected, when the biliary stent was removed. Owing to the refusal of transfusions, it was important to minimize the bleeding.

The most common indication for biliary stent insertion is gallstones in the common bile duct. Other indications include stenosis or obstruction of the common bile duct due to various benign or malignant conditions. In this case, the ERCP showed a normal cholangiogram and the indication for stent insertion can be discussed/debated.

This case highlights the importance of proper imaging diagnostics, which was the reason for the surgical backup ready for laparotomy. The case further confirms the importance of a multidisciplinary approach to biliary stent complications. Still, biliary stents should be placed on relevant indications.
